# Heart rate variability is more sensitive to stress than heart rate in specialist police undergoing selection

**DOI:** 10.1371/journal.pone.0317124

**Published:** 2025-01-24

**Authors:** Colin Tomes, Ben Schram, Elisa Canetti, Robin Orr

**Affiliations:** 1 Faculty of Health Science and Medicine, Bond University, Robina, QLD, Australia; 2 Tactical Research Unit, Bond University, Robina, QLD, Australia; Amazonas State University, BRAZIL

## Abstract

Police tactical group (PTG) officers respond to the most demanding and high-risk police situations. As such, PTG personnel require exceptional physical fitness, and selection for employment often evaluates fitness both directly and indirectly. While heart rate (HR) is often used to measure physical effort, heart rate variability (HRV) may be a valuable tool for measuring stress holistically. The primary aim of this research was to investigate whether HRV was more sensitive than HR at monitoring workload during key PTG selection activities. As aerobic fitness is associated with workload during these tasks, a secondary aim was to investigate relationships between HRV, HR and aerobic fitness during the same tasks. The relationships between HRV (percentage of adjacent R-R intervals varying by 50% or more; pRR50%) and HR, as measured by ambulatory electrocardiograms obtained during a specialist police selection course, as well as aerobic fitness, as determined via total shuttles completed on the 20-meter multistage fitness test (MSFT; ‘beep test’), were investigated. This study included a cohort of six male PTG candidates (n = 6) undergoing selection. As illustrated by a time-series plot, HR values were generally unremarkable, but HRV values were potentially depressed, and tentatively indicated overstress when count data from consecutive short-term analyses were derived. The MSFT was significantly, positively, correlated with pRR50% (ρ (6) = 0.812, p = 0.050, Fisher’s z = 1.132). The MSFT and nonlinear HRV, frequency domain HRV, and HR were not significantly correlated. When assessed by linear regression, neither HRV nor HR were predicted by MSFT score. These findings indicate that HR alone is likely not sufficiently sensitive to provide detail on the stress response of candidates undertaking essential tactical tasks that combine physical stressors with cognitive load in adverse conditions. HRV analysis may provide additional insights regarding candidate suitability, particularly during dynamic and multifaceted assessments, though the causal direction of the relationship between HRV and aerobic fitness remains unclear.

## Introduction

The law enforcement professional is consistently ranked as one of the most physically demanding occupations [1–3]. Police officers, in the execution of their duties, may need to cover distances quickly over ground and various terrain, negotiate past obstacles, maintain balance, subdue suspects, lift, climb, and push or pull substantial weights potentially with no advanced notice [4, 5]. Furthermore, these physical tasks must often be performed whilst carrying an external load, often exceeding 10 kg [1] and over shifts that may last between 10 and 12 hours [6]. As a result, police officers are exposed to great physiological and psychological stress [7]. Therefore, not only fitness, but resilience may be crucial for law enforcement personnel to complete their occupational tasks both safely and effectively throughout their careers, which may span decades [8, 9].

Within law enforcement, incidents and operations that carry the highest expectations of risk are assigned to police tactical groups, or PTGs, when possible. PTGs may differ in their approach to these high-risk scenarios from conventional law enforcement practices. Because of the additional challenges associated with tactical police duties, high levels of fitness are required and frequent exposure to stressful situations is expected [7, 8]. As such, the processes in place to select PTG members must impose physical challenge and high stress in occupationally relevant contexts [10, 11]. PTG leaders and selection course assessors test and measure candidates for general aptitude, tolerance of uncertainty, leadership, and responses to adversity, in addition to physical fitness [7, 12]. As an example, one exercise typical across many tactical selection courses is land navigation or orienteering [13, 14]. These challenges require candidates to plan and manoeuvre through a given territory to reach designated navigation points without aids other than a map and compass. Land navigation drills are known to be physically demanding, but to complete an orienteering challenge successfully, candidates must accurately calculate details of their route plans, recall their step counts, and measure their cadence [14]. Beyond this, climate and other regional conditions may be selected by course administrators for adversity, such as extreme heat or cold, harsh terrain and over extended time periods, such as overnight. Land navigation exercises may also be scheduled adjacent to other physical and cognitive tasks, and candidates may not be permitted to rest during the event of after concluding. Therefore, PTG candidates are likely at an increased risk of allostatic load sources during selection [15, 16].

Allostatic load, first posited by Bruce McEwen and colleagues in 1998 [17, 18], describes insufficient adaptation, or failure of adaptive mechanisms in response to sustained stress loads that lie beyond the individual’s capacity. Allostatic load is an integrative and holistic framework for understanding stress responses to uncertainty, inclusive of both physical and psychosocial stress sources [19]. Given the wide variety and high intensity of stress exposures encountered by law enforcement personnel, the quantitative assessment of allostatic load may be of high value to understanding psychophysiological states of these personnel [20].

Heart rate variability (HRV), the computational assessment of fluctuation and oscillation of heart activity over time, arises from the relationships between the heart’s own regulatory mechanisms, such as the cardiac conduction circuits, as well as mechanisms extrinsic to the heart, namely the sympathetic (SNS) and parasympathetic (PNS) nervous systems [21, 22]. The SNS is primarily responsible for the "fight or flight" response, increasing heart rate and decreasing HRV through catecholamine and cortisol release, which can indicate heightened stress or arousal. Conversely, the PNS promotes a "rest and digest" state, leading to decreased heart rate and increased HRV. Given the relatively rapid function of the PNS relative to SNS as related to cardiac activity modulation [23], time domain measures such as root mean square of successive differences (RMSSD) and percentage of adjacent R-R intervals varying by 50 milliseconds (pRR50) may be particularly demonstrative of these physiological shifts over ECG evaluations under 24 hours [21, 24]. In addition to these conventional measures, fractal self-similarity measures such as alpha and beta exponents have gained attention for their ability to characterize the complexity of heart rate dynamics [25]. These measures reflect the underlying physiological processes influenced by both the SNS and PNS, providing insights into the balance between them [22]. Importantly though, while HRV can indicate shifts in autonomic activity, it does not necessarily assess the balance between SNS and PNS activity since both systems can be simultaneously active [26].

HRV may therefore be utilised as a non-invasive tool holistically quantifying stress in PTG or similar high-stress populations [27], particularly while engaging in strenuous operations or training [28–30]. While HRV has shown to trend positively with cardiometabolic fitness [31], it may not be a concern in specialist police units, and is already assessed heavily. However, additional stress sources that may also impose allostatic load, such as cognitive tasks [15, 32], should not be overlooked. Likewise, age is known to adversely influence HRV [33], but may also not be a relevant confounder in the PTG setting. HR, while easily obtained through a variety of means as a measure of exertion [34], may not independently signal excessive stress, and indeed, some research on critical police tasks such as marksmanship find a relationship with HR [35] and others do not [36]. Conversely, more precise laboratory measures for ANS measurement, such as muscle sympathetic nerve activity or functional magnetic resonance imaging (fMRI), are not practical in the austere environments associated with PTG unit training and operations. Therefore, HRV may be the most feasible method for stress quantification in the PTG setting [37], with previous research indicating that a variety of HRV measurement methods relate to PTG or other specialist police tasks [38, 39].

In addition, while extant studies at the meta-analytical level have concluded that aerobic fitness is a viable predictor of injuries sustained during tactical training, specifically initial entry training [40], the specific underlying mechanisms still remain unclear. While numerous contributors are likely involved regarding the relationship between training injury and physical fitness, candidates or trainees with higher levels of aerobic fitness are able to maintain a given physical workload with less effort than peers who are not as physically fit [41, 42]. The consequent reduction in relative load as fitness levels increase may have a protective effect. Indeed, research conducted in the military setting identified such a protective relationship also extended to vigilance and cognitive tasks [43]. However, whether this phenomenon is also true for PTG personnel undertaking tasks of both a physical and cognitively taxing nature (e.g., assessed and timed navigation tasks) remains unknown. While a recent systematic review did find relationships between HRV and aerobic fitness [44], this finding was not uniform. Indeed, one study [45], whose participants (emergency response workers) were measured to have an average VO_2max_ of 51 ml/kg/min, did not find a relationship between oxygen uptake and HRV, indicating a ceiling effect may occur, limiting the positive relationship trend between HRV and aerobic fitness as VO_2max_ approaches very fit to elite levels. However, the participants in the above study were evaluated as they recovered from a 24-hour shift, and as such their time off-duty could have imposed minimal cognitive demand [45]. This is notable given that PTG candidates typically attain very high levels of fitness but may also frequently perform tasks that are both cognitively and physically challenging [8]. Thus, analysing HRV may be an optimal approach to describe this dynamic with detail in situ [27].

The primary aim of this research was to investigate whether HRV was more sensitive than HR at monitoring stress holistically during critical PTG selection course activities. Given that aerobic fitness is associated with workload, a secondary interest in this study was to assess relationships between HR, HRV, and aerobic fitness during the same selection activities. It was hypothesised that HRV would be more sensitive to responses that may indicate allostatic load than HR. Finally, HRV was expected to be more closely related to physical fitness than HR throughout PTG selection activities, as HRV assessment may more effectively indicate holistic psychophysiological response.

## Materials and methods

The present study was conducted at an Australian State PTG facility in Autumn of 2022. A cohort design was utilised, and a combination of retrospective and prospective data were obtained an analysed. HRV was measured continuously using wearable electrocardiogram (ECG) devices for the entirety of an approximately five-hour land navigation exercise. Temperature ranged from 24.2–34.4°C and relative humidity ranged from 60–75%. Aerobic fitness data were derived from candidate records of the multistage fitness test (MSFT) and were documented as level, shuttles. All procedures were conducted in accord with the Declaration of Helsinki of 1964 and its later amendments. Candidates provided their informed written consent, and the PTG unit provided permission for publication of this work. The research protocol was approved by the Bond University Human Research Ethics Committee (2019–022 amendment 2). Participant data were anonymized upon collection, with no personal identifiers attached to any data records. All data were identified by a random participant number only.

An initial cohort of 18 male Australian State Police Officers presented for a one-day physical training assessment and selection course. While female officers were permitted at selection and were therefore eligible for inclusion in this study, none had applied or presented for selection. Attrition during the one-day assessment was high; 12 candidates initially entering the course either voluntarily withdrew or did not meet physical fitness standards. Of those 18, only six individuals were eligible to participate in the additional two-day selection course on which this study was based. All candidates eligible for the two-day selection course were eligible for inclusion in this study. There were no exclusion criteria, and all available personnel were recruited and consented. Individual anthropometric data were not available as per a privacy agreement with the PTG, however, all candidates disclosed that they were taking no medications for cardiovascular, renal, or respiratory conditions as a requirement of participation in this study. Combined data can be found in Table 1 and includes anthropometrics, load carriage weights, and aerobic fitness data.

**Table 1:** Anthropometric, load carriage and fitness data of Australian PTG candidates.

Value	Body Mass (Kg)	Height (cm)	Age (Years)	Land Nav Equipment Mass (Kg)	Pack March Equipment Mass (Kg)	BMI (Kg/m^2^)	MSFT (Total shuttles)
Mean	93.63	180.17	30.67	12.24	11.95	28.88	84.17
SD	8.83	5.98	2.94	0.14	1.32	2.92	7.22
Median	94.78	181.00	31.00	12.24	11.96	29.22	81.00
Range	20.50	21.08	14.00	8.00	0.20	8.47	1.9

body mass index (BMI), multistage fitness test (MSFT). Range is reported as the difference between maximum and minimum values.

For this study, height was self-reported; self-reported anthropometrics have been utilised as reliable metrics in research in previous literature in law enforcement populations [46]. It should be noted that while HRV may be influenced by factors such as age, body mass, and potentially body mass index (BMI), these data limitations are not uncommon in the population of interest [47]. Further, the summarised data presented demonstrate little variance (Table 1). The aerobic fitness data were collected from the 20-m Multistage Fitness Test (MSFT) conducted approximately two weeks prior to the selection course following protocols previously described in the literature [48–50]. Final levels and stages were converted to total number of shuttles completed for the analysis as opposed to conversion to VO_2max_ given limitations in these predictions [51].

The two-day selection course consisted of additional physical training activities, but also essential specialist police task training and assessment. Activities included orienteering, firearms manipulation, threat de-escalation, load carriage, and casualty evacuation. The land navigation (orienteering) exercise consisted of a sequence of navigational points candidates located and travelled to on foot utilising orienteering methods. This task was not only physically, but cognitively demanding and is a known source of attrition for many tactical professions [52–55]. Further details of the selection activities can be found in Table 2.

**Table 2:** Description of selection course serials and start times.

Serial	Description	Start Time (Duration)
Briefings and equipment issue	Candidates were briefed on expectations and role of the PTG. Standardised equipment was provided and any individual items were inspected for approval.	0705 (1:50)
Written examination	Multimodal assessment of land navigation (orienteering) principles.	0850 (0:25)
Administration	Mobilisation, review, and further explanation of expectations for the land navigation exercise.	0930 (0:35)
Land Navigation Preparation	Candidates were provided time to plan their routes and mark their maps before embarking.	1005 (0:20)
Land Navigation Exercise	Orienteering assessment of navigation over various terrain using only a supplied map, compass, and grease pencil.	1018 (5:00)
Administration	Mobilisation, hands-on assessment, and written safety/proficiency check.	1547 (2:50)
Load Carriage/Pack March	Various load carriage and transportation of stores at self-selected pace. Candidates proceeded for undisclosed time/distance.	1850 (-)

All candidates were supplied EQ02+ LifeMonitor (Equivital™, Cambridge, UK) wearable monitoring harnesses to capture ECG signals throughout the selection course. Each harness was individually fitted to each candidate to ensure electrode contact points were secured. Previous research indicates the Equivital system is comparable in terms of validity and reliability to the gold-standard Holter ECG monitor when artifact levels are low (<20%) [56]. Physiological measures were observed through accompanying software (EQ View Pro, Equivital™, Cambridge, UK, and LabChart 8 Pro, ADInstruments, Sydney, Australia). After data collection was complete, ECGs were examined by the authors in combination with automated rhythm irregularities checks that occur during data processing when ECG files are imported into the LabChart Software (version 8). ECG complexity, a measurement of QRS complex quality, was set in LabChart to an acceptable range of 1.0 to 1.5. Acceptable R-R intervals (RRI) were established as those between 272ms and 1600ms. This is equivalent to an HR between 220 bpm and 37.5 bpm. Any RRI outside of this range was manually analysed and included or excluded from the HRV report based on the visual features of the ECG.

HRV measurements were assessed under both long-term and short-term ranges. For the purposes of correlation and regression analyses, the more stable, long-term value was utilised, as the task of interest was over five hours duration. The short-term analyses were compiled as consecutive 5-min samples plotted as a time series to demonstrate the most precise moment-to-moment changes in HR and HRV with acceptable validity. Analysis of the temporal data was informed by the work of Buchheit and Hopkins [57, 58], in which the mean and standard deviation (SD) of each participant’s HRV values are designated as thresholds. The total count of 5-minute samples with HRV values below or above the generated threshold (±½SD of the participant’s long-term mean) were also considered for regression. Using within-participant thresholds controls for the highly individual nature of HRV and its volatility while still permitting meaningful analysis of the entire cohort [59].

For inferential analysis, HRV was assessed by time-domain (percentage of adjacent R to R waves varying by at least 50ms across entire sequence of recording; pRR50% and root-mean square of successive differences; RMSSD), nonlinear (Poincare plot SD1 and SD2), and frequency domain (high frequency; HF and low frequency; LF) measures. The ECG recording window of interest began at approximately 0730 and ended at approximately 2000 local time. This timeframe comprised the entirety of the land navigation exercise, which consisted of an average traversed distance of 9.07 km over mostly even, but hazardous terrain in environmental conditions ranging from 24.2–34.4°C and relative humidity ranged from 60–75%. Short-term analyses were conducted via the method described above.

Descriptive statistics, including the Shapiro-Wilk test for normality, were obtained, and considered prior to any inferential statistical approach. Distribution plots were also analysed visually by variable. Spearman’s rho was selected for correlation analyses, as it is appropriate for non-parametric analyses [60].

## Results

**Fig 1:**
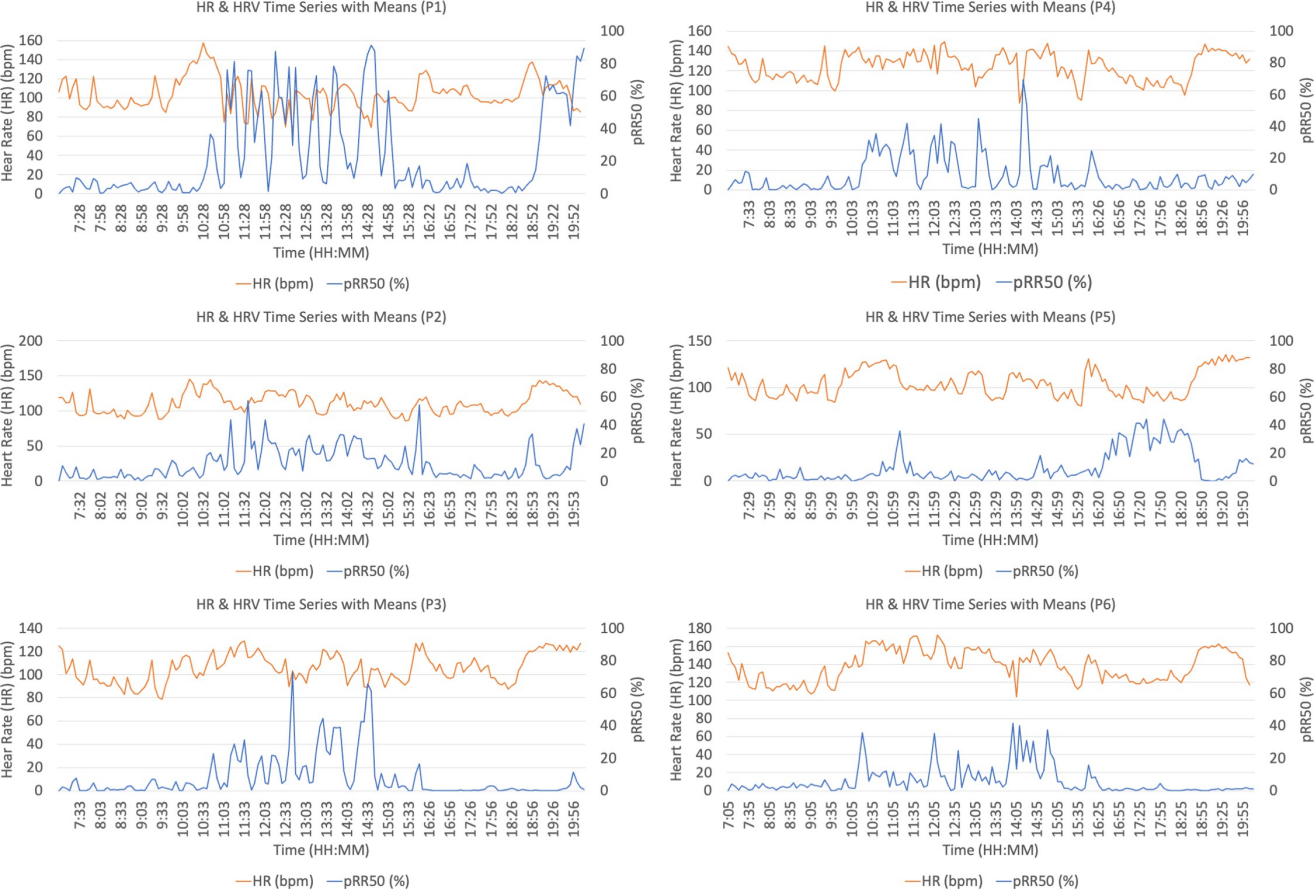
Comparison of heart rate (HR) and HRV values by participant (P1 –P6). Values demonstrate that while HR values may appear unremarkable, HRV values can fluctuate and indicate stress and workload levels more precisely.

All collected data fell within the ‘low’ range for artifact presence (<20%) [56]. All six candidates successfully completed the land navigation event. Attrition events began occurring at approximately 1945–2000 hours, leading to the conclusion of the data collection window. Time-series plots of HR and HRV (pRR50%) are shown below in Fig 1. Visual inspection demonstrates the difference in fluctuations between HR and HRV during training. Count data from the temporal analysis of the land navigation event (1005–1547) were dervied and total values above and below >½SD are reported along with summarised MSFT, HR, and HRV values in Table 3.

**Table 3:** Summarised MSFT, HR, and HRV values.

	MSFT (s)	MSFT Shuttles	Mean HR (bpm)	pRR50%	SD1 (ms)	SD2 (ms)	HF (nu)	LF (nu)	RMSSD (ms)	5-min counts <½SD (pRR50%)	5-min counts >½SD (pRR50%)
Mean	474.33	84.17	121.82	11.40	93.35	147.21	53.50	38.25	132.02	36	21.7
SD	24.15	7.22	19.93	6.34	42.61	53.58	13.09	17.11	60.28	13.8	10.4
Median	466.50	81.00	118.10	9.23	105.12	149.45	56.88	33.16	148.70	35	24.5
Range	66.00	19.00	57.09	17.53	123.21	154.55	37.07	46.40	174.30	36	29

MSFT: Multistage Fitness Test, HR: Heart Rate, bpm: beats per minute, pRR50: percentage of R-to-R intervals varying by at least 50ms, SD1: Geometric Standard Deviation 1 (X-axis), SD2: Geometric Standard Deviation 2 (Y-axis), HF: High-frequency, LF: Low-frequency, RMSSD: root-mean square of successive differences, SD: standard deviation. Range is reported as the difference between maximum and minimum values. Values reported are for the entire duration reported.

After conversion from raw score to total shuttles, the MSFT scores were substantially skewed. For this reason, further analysis conducted after correlation tests were conducted using a transformation of total MSFT shuttles. The transformation that best achieved tolerable skew, kurtosis, and normality was the log10 of the identity. Further details regarding this method are described by Tabachnick and Fidell [60].

**Fig 2:**
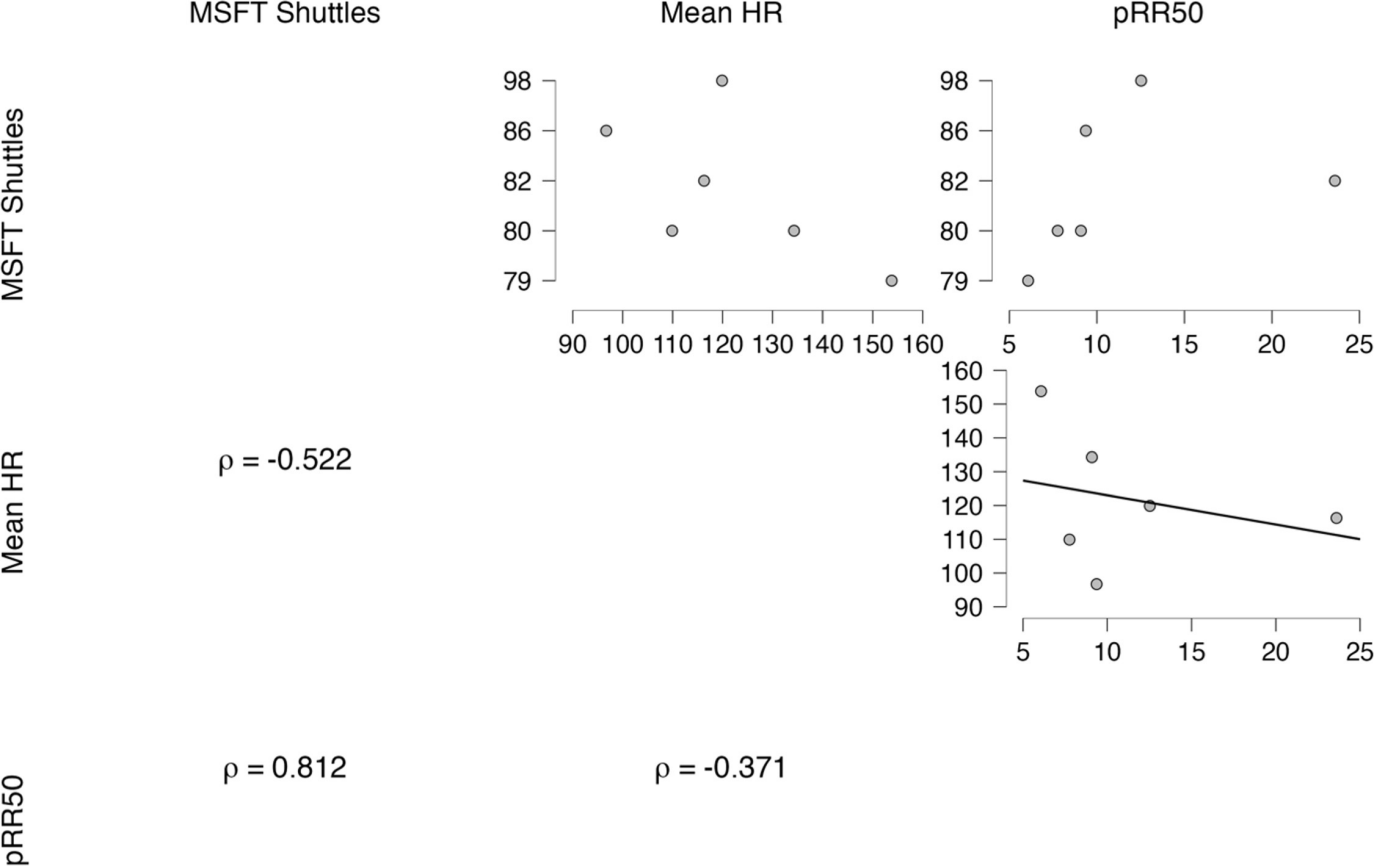
Triangular matrix of Spearman’s rho correlation scatter plots. Visual description of the relationships between HR, HRV, and MSFT time. Elements along the diagonal are equal to the identity.

Regarding the results of the correlation analysis, the percentage of pRR50 intervals was significantly and positively correlated (ρ (6) = 0.812, p = 0.050, Fisher’s z = 1.132) with the MSFT (total shuttles completed without transformation). HR (ρ (6) = -0.522, p = 0.288) and other HRV metric results were not significantly correlated with the MSFT. Further details regarding the correlation analyses can be found in Fig 2.

The HR and HRV data did not require transformation. Results of the linear regression models can be found below in Table 4:

**Table 4:** Regression analysis results.

	R^2^	*F* Statistic	*p*-value
HR by Log10 MSFT	0.113	1,4 = 0.512	0.514
pRR50% by Log10 MSFT	0.030	1,4 = 0.124	0.742
5-min counts <½SD by Log10 MSFT	0.034	1,4 = 0.139	0.728
5-min counts >½SD by Log10 MSFT	0.010	1,4 = 0.039	0.854

MSFT: Multistage Fitness Test, HR: Heart Rate, bpm: beats per minute, pRR50: percentage of R-to-R intervals varying by at least 50ms, SD: standard deviation.

Neither the linear model for prediction of raw HRV (pRR50%), count HRV data, nor the model for prediction of HR reached statistical significance (*p >*0.05).

## Discussion

The primary aim of this research was to investigate whether HRV was more sensitive than HR at monitoring workload during key PTG selection activities. As aerobic fitness is associated with workload during these tasks, a secondary aim was to investigate relationships between HRV, HR and aerobic fitness during the same tasks. The hypothesis that HRV would be more sensitive to allostatic load responses than HR was potentially confirmed. The hypothesis that HRV would be more strongly related to aerobic fitness than HR during selection activities was tentatively confirmed but could not be completely verified.

HRV, specifically the pRR50 index, demonstrated that even when physical exertion may be moderate to low, as indicated by HR (average HR: 121.82±19.93 bpm, Table 1), HRV shows a more precise picture of potential stress or workload, as HR values were generally unremarkable. HRV values, however, were generally suboptimal and suggest overstress, in that while depression of HRV, specifically pRR50 intervals will regularly drop to zero or near-zero levels during exercise (mean pRR50: 11.40±6.34%, Table 1), the literature generally describes exercise protocols of high intensity (~75% HR_max_) producing these responses [61]. Given the low HRV observed in this study without commensurate elevation in HR suggests that either additional factors like cognitive load and uncertainty intolerance were present to drive a greater stress response than HR would indicate, a level of exhaustion was present, or a combination of these factors contributed to the overall psychophysiological picture [62–64]. Indeed, the potential value of HRV, particularly in tactical settings where a multitude of stress sources are present, is the integrative nature of the assessment, with HRV being both sensitive and individualized.

Nonetheless, it is not unreasonable to posit that cognitive load likely explains why the land navigation event resulted in disparity between HR and HRV measures [13, 14, 65, 66]. As stated, none of the candidates approached heart rates that might be considered near maximal during the navigation exercise [49]. Essentially, the submaximal physical nature, yet high overall stress induced by environmental conditions, pressure to succeed, cognitive challenge, and imposition of uncertainty could not be discriminated by HR alone. Fluctuations in HRV, however, which is sensitive to cognitive load in addition to physical load, were more effective measures to indicate potential overstress: an average HR of 138 bpm (the highest observed in this study) is not anomalous, however, pRR50% values below approximately 3% are potentially of concern [61, 67]. The count data derived from the time-series plots (Fig 1) further emphasize this finding; candidates averaged 36 total incidences of 5-min HRV measures under half a standard deviation from their mean, or about 180 minutes. This was over half the entire duration of the land navigation event. If candidates were generally of high fitness, as the data would support, the physical load alone of the navigation exercise may not have resulted in sufficient physical strain for fitness levels to stratify HR during the event. Conversely, the cognitive load may have been more taxing for the candidates in this selection course [68].

The results show that during the PTG task battery, HRV trends with aerobic fitness, but HR does not. However, the causal relationship could not be evaluated definitively in this study. Indeed, while the effect size (Fisher’s z) of 1.132 corresponds to an approximate correlation coefficient of about 0.81 (using the inverse transformation), indicating a strong positive correlation, the *p*-value of exactly 0.050 may be interpreted as equivocal. A further caution in the interpretation of the results presented here is that no other index of HRV (nonlinear, RMSSD, frequency-domain) reached statistical significance in correlation analysis. It may be possible that high aerobic fitness attenuates HRV depression when under stress from multiple sources, but the linear regression model results do not support this conclusion. As such, it is likely that aerobic fitness, while important, may be limited in its contribution to overall success in specialist police selection. Factors to which HRV is sensitive, such as cognitive load and emotional strain, should also be considered. Indeed, the ANS, a primary driver of HRV, is responsive to a wide variety of stimuli, and therefore determining a singular explanatory influence on HRV is unlikely. While greater aerobic fitness may potentiate stress, there may be a ceiling above which additional aerobic capacity lends no further advantage. Indeed, findings from a recent systematic review also posit this hypothesis [44]. Namely, the study by LyytikÄInen and colleagues also did not identify a relationship between HRV recovery and aerobic fitness following a 24hr shift in a cohort of emergency services personnel [45]. However, other research does suggest aerobic fitness can support vigilance in a similar context to that described here [43]. Future studies may aim to clarify the dose-response curve with larger cohorts to characterise the point at which aerobic fitness may no longer attenuate psychosocial stress factors in tactical settings. It may also be possible that the natural volatility of HRV between individuals limits the extent to which relationships between HRV and physical fitness may be generalized [21, 33]. Lastly, while age was unlikely a meaningful confounder in this study, the influence of aging on HRV is known to be more pronounced in men than in women [69]. Given the large preponderance of men in law enforcement, this may be a key consideration in future works.

This research may be of interest to individuals across tactical professions interested in objective psychophysiological assessment. The findings described in this study indicate that HR alone is likely not sufficiently sensitive, especially in small cohorts, to provide detail regarding how aerobic fitness may be associated with performance during essential tactical tasks that combine physical stressors with cognitive load in adverse conditions [27]. HRV analysis may provide additional insights regarding candidate suitability to unit leadership and directional staff, as candidates were more stratified in the HRV responses than their HR responses. The approach presented in this report may provide a framework for other high-stress occupations that must contend with small recruit populations, high attrition, and multifactorial stress exposures seeking means of objectively assessing and stratifying individuals, even when traditional measures of fitness and exertion are not clearly delineated.

### Limitations

The chief limitation of this study is small sample size (n = 6). While this value represents the entire roster of selection candidates at the time the study was conducted, the generalizability of the reported findings is severely limited. Additional research with larger cohorts may enhance the robustness of this study’s conclusion. While beneficial for internal validity of the analyses, the lack of any female participants presents another limitation to the wider applicability of this work. Such research specifically in female law enforcement personnel is warranted. Further limitations include the lack of an objective measure of cognitive load. While applying such a measure in context may prove impractical given the substantial distances and remote terrain in which the candidates were operating, further studies may attempt to quantify cognitive load in this specific occupational context, providing valuable data to substantiate the conclusions drawn in this work. While the sample size is not unusual in the population of interest, in combination with the generally high and uniform aerobic fitness testing results, the precise nature of the relationship between HRV and aerobic fitness may not have been fully characterised. Additionally, while the MSFT is known to be a reliable and valid measure of aerobic fitness, it may not have been a true measure of maximal aerobic capacity in this context. Future research may utilise lab-based measures of aerobic capacity, rather than field measurements to further refine the relationships between HRV and aerobic fitness in tactical personnel.

## Conclusions

This study provides tentative, but still valuable insights into the monitoring of HR and HRV among a cohort of specialist tactical police candidates. The findings confirm the primary hypothesis that HRV exhibits a higher sensitivity to allostatic load responses compared to HR. However, the hypothesis positing a stronger relationship between HRV and aerobic fitness than between HR and aerobic fitness during selection activities was not entirely verified. The diversity of stress sources candidates encountered, including submaximal physical demands, environmental conditions, the pursuit of success, cognitive challenges, and the imposition of uncertainty underscores the limitations of HR as a standalone measure. In contrast, the nuanced fluctuations in HRV, reflecting sensitivity not only to physical load but also cognitive load, emerge as more effective indicators of potential overstress in these scenarios. In consideration of future research, the analysis of HRV holds promise in providing additional information pertinent to evaluating candidate suitability. As the variety and intensity of stressors continue to shape the demands on tactical police candidates, a comprehensive assessment encompassing HRV could offer a more holistic understanding of their psychophysiological responses. In summary, this study underscores the significance of HRV as a complementary metric alongside HR in gauging psychophysiological responses among specialist tactical police candidates. While further research is warranted, specifically recruiting larger cohorts over greater lengths of time, the current findings establish a more comprehensive approach to enhancing the monitoring and assessment protocols within tactical training contexts.
